# Group‐Specific Nonlinear Mixed Effects Models for Longitudinal Data With Realignment of Time

**DOI:** 10.1002/sim.70759

**Published:** 2026-10-01

**Authors:** Ziyue Liu, Sujuan Gao

**Affiliations:** ^1^ Department of Biostatistics and Health Data Science Indiana University School of Medicine Indianapolis Indiana USA

**Keywords:** longitudinal, mixed effects, nonlinear, prediction, survival

## Abstract

We propose group‐specific nonlinear mixed effects models for longitudinal data whose indexing time is right‐aligned to specific events, modeled as the time preceding those events. These models are motivated by dementia cohort studies that examine nonlinear trajectories of longitudinal cognitive test scores in late life, with separate trajectories for individuals with and without dementia in the period leading up to death. The model framework includes three sub‐models: one for group membership, which represents dementia status in our motivating data, one for time to the indexing events, and one for the longitudinal outcome. Group membership is modeled using logistic regression or random forest. Conditional on group memberships, time to indexing events is modeled using accelerated failure time (AFT) models. Given both group membership and time to the indexing event, longitudinal data are modeled using nonlinear mixed effects models. By modeling and integrating out the time to indexing events, individuals whose indexing events have not been observed can be incorporated into the models. Stochastic approximation expectation maximization (SAEM) algorithms are adopted to maximize the likelihood for parameter estimation. In addition to modeling group‐specific longitudinal trajectories aligned to the index time, the proposed models can also be used to predict group memberships or improve the prediction of time to the indexing events. Simulation studies are conducted to evaluate finite sample performance. An application to data from a dementia study is used for illustration.

## 1 Introduction

One fundamental challenge in modeling longitudinal data from observational cohort studies is the alignment of time. In randomized clinical trials, longitudinal measurements are typically anchored to a clearly defined starting point, such as randomization or the initiation of an intervention. This consistent alignment facilitates straightforward estimations of the longitudinal trajectories with associated factors and meaningful comparisons across groups of individuals. In contrast, observational cohort studies enroll participants at varying and sometimes arbitrary time points in the life course and thus lack a uniformly defined starting point. In these studies, time is frequently indexed using chronological age, which assumes that longitudinal outcomes depend primarily on age and that individuals are comparable at the same ages. However, this assumption may be inappropriate when the processes of interest are more closely tied to specific events than to age itself. Realigning time relative to a meaningful indexing event, such as disease onset or death, provides an alternative framework that can better capture the underlying dynamics of change in the time period before or after the index event. This approach is particularly important when studying late‐life processes, where trajectories may accelerate or shift in proximity to the event. By aligning individuals according to a biological or clinical milestone, rather than chronological age, researchers can reduce heterogeneity, improve interpretability, and more accurately characterize patterns of change associated with the event of interest. In many observational cohorts, however, some participants have not yet experienced the indexing event by the end of follow‐up. Excluding these participants will limit the inference scope to subjects with complete data only, whereas including them requires modeling approaches that account for their unobserved event times while fully utilizing their available longitudinal measurements.

The second challenge in modeling longitudinal data from cohort studies arises from heterogeneity among individuals. In many studies, different subgroups of individuals may follow distinct longitudinal trajectories. For example, in dementia research, individuals who develop dementia often exhibit greater cognitive decline than those who remain cognitively normal. When disease status is observed for all participants, it can be incorporated into the models, such as group‐based trajectory modeling [1] and growth mixture modeling [2]. However, in some cohort studies, disease statuses are determined through extensive cognitive testing and clinical evaluations, thus may remain undetermined for some individuals, which creates additional complexity when modeling group‐specific trajectories. The third challenge is accommodating nonlinear longitudinal trajectories. Biological processes and disease progression are rarely linear, and flexible models are often required to capture nonlinear patterns over time. However, incorporating nonlinear trajectories can introduce substantial computational complexity and methodological challenges.

The primary contribution of our proposed method is a modeling framework that addresses all three challenges by incorporating group‐specific, nonlinear models with time realignment, while accommodating participants whose time‐to‐event or group membership is unobserved. Our motivation for developing a new modeling framework is driven by longitudinal data from dementia studies. In many large epidemiologic cohort studies focused on dementia in the elderly, cognitive scores are typically collected at regular time intervals. A particular research objective is to compare the cognitive trajectories between individuals diagnosed with dementia and those who remain cognitively normal in late life in the period leading to death. Importantly, cognitive decline is not exclusive to dementia; cognitively normal individuals often experience measurable decline in the years preceding death. This raises a key question: do individuals with dementia exhibit a steeper or different pattern of decline during this terminal period compared to those without dementia? Addressing this question requires models that can accurately capture changes in cognition as a function of time‐to‐death, rather than simply chronological age. Understanding these distinct patterns of late‐life cognitive decline is critical. For example, linking trajectories of cognitive change to underlying neuropathology will offer a unique opportunity to disentangle disease‐specific processes from normal aging and terminal decline.

In our motivating data, the longitudinal measurements are cognitive scores, the indexing event is death, and the group membership is whether an individual was diagnosed with dementia before death. Figure 1 displays longitudinal cognitive scores from African American participants in the Indianapolis‐Ibadan Dementia Project, using three different methods of time alignment [4]. Figure 1a shows data from individuals diagnosed with dementia by the time of death, while Figure 1b shows data from those without a dementia diagnosis at death. Both the raw data and the fitted mean functions, based on sigmoidal functions as described in Capuano and Wagner [3], reveal differential longitudinal patterns between the two groups when scores are aligned by time‐to‐death. In contrast, when the cognitive scores are aligned by time since baseline or by chronological age, as shown in Figure 1c–f, these patterns are much less discernible by visual examination.

**FIGURE 1:**
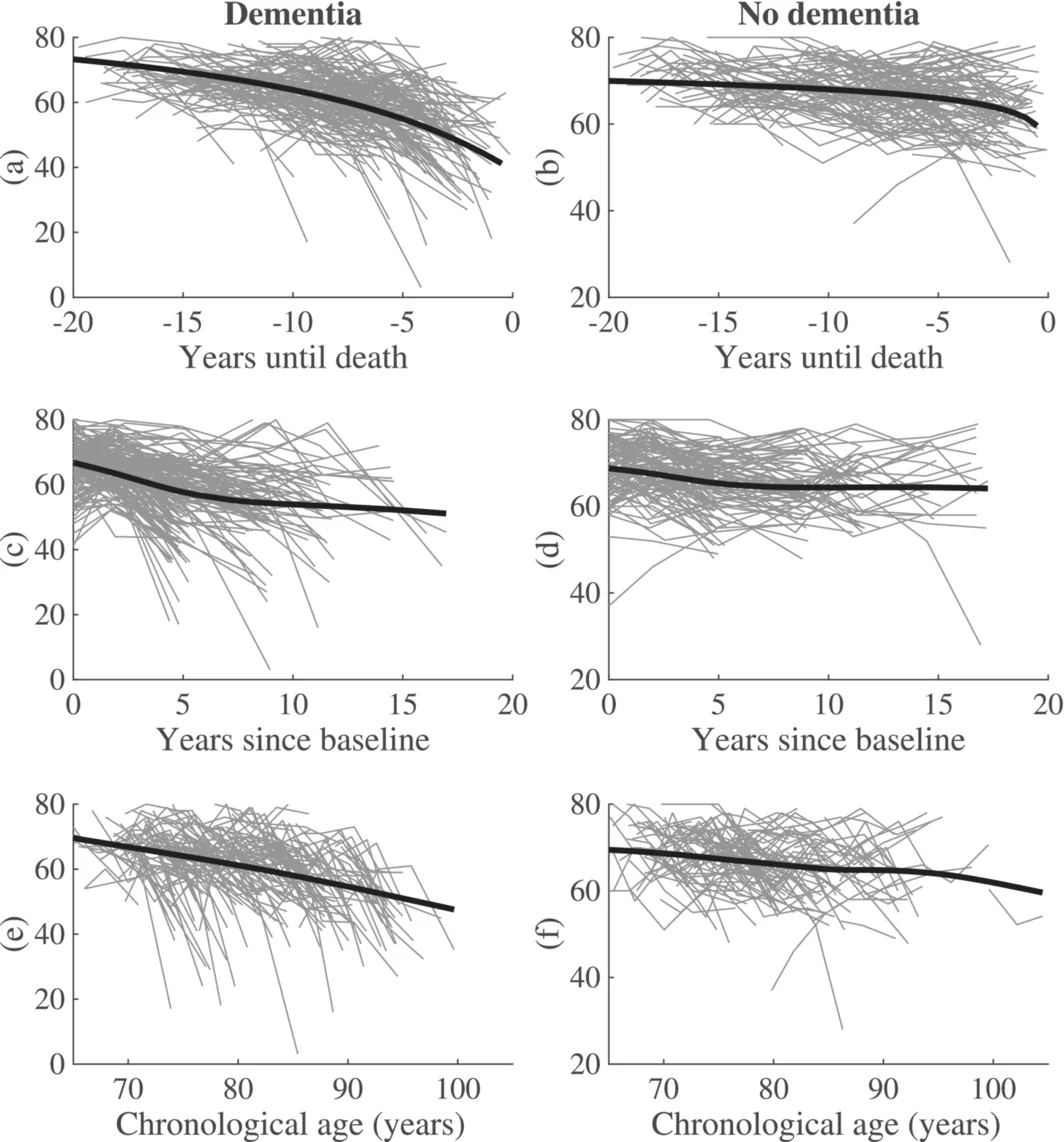
Longitudinal cognitive scores for individuals who died with and without a dementia diagnosis. The top row figures are aligned at death as years until death, the middle row aligned at enrollment as years since baseline, and the bottom row aligned by the chronological age. The thin gray lines are the raw data while the thick black lines are the estimated mean functions. The top row means were estimated using the models of Capuano and Wagner [3], while the middle row and bottom row means were estimated by monotonic spline smoothing while ignoring the within‐individual correlations.

In the literature, longitudinal data from the aging processes have been widely studied. Most approaches were based on left‐aligned time to certain events such as time since enrollment or chronological age. For example, one approach was to first extract the longitudinal characteristics from a time period following enrollment, and then use the extracted characteristics to predict dementia in subsequent time periods [5, 6, 7]. A second approach was to use longitudinal markers as time‐dependent risk factors [8, 9, 10]. A third approach was joint modeling of longitudinal data with time‐to‐event outcomes [11, 12] or continuous cognitive measures [13]. A fourth approach was to use the longitudinal data to define a biological age, based on which survival models were developed to predict dementia [14, 15]. Dementia onset time or death time had also been adopted to realign the longitudinal data [3, 6, 16, 17, 18, 19, 20]. However, these are only applicable to individuals with observed indexing events but not to those whose events have not been observed.

In this article, we propose a class of group‐specific nonlinear mixed effects models with realignment of time that are capable of dealing with all three challenges we described above. The first model component is the longitudinal model conditional on both the group memberships and the indexing event time. To handle the nonlinear trajectories that are commonly encountered, we propose to adopt parametric nonlinear mixed effects models like in Capuano and Wagner [3]. These models offer sufficient flexibility while utilizing a small number of parameters. The second component is a model for group membership, which may depend on covariates. The third component is a survival model for the time to the indexing event, where we adopt accelerated failure time (AFT) models. By jointly modeling time to the indexing event and eventually integrating it out, we will be able to incorporate individuals whose indexing events have not been observed. Parameter estimation and inference are conducted using a likelihood‐based approach. Stochastic approximation expectation maximization (SAEM) algorithms are adopted to maximize the likelihood [21, 22]. Hypothesis testing is performed, and confidence intervals are constructed based on the maximum likelihood estimates of the parameters and their asymptotic variances. Model selection is based on testing additional parameters for nested models and the Akaike information criterion (AIC) for non‐nested models.

The remainder of the article is organized as follows. Section 2 introduces the proposed models. Section 3 presents a simulation study. Section 4 provides results from the motivating data example shown in Figure 1. Discussions follow in Section 5.

## 2 The Proposed Method

### 2.1 The Proposed Model

#### 2.1.1 The Overall Model

For the ith subject, let $z_{i}$ be a column vector of baseline covariates, $I_{i}=1,\cdots \;,K$ indicate the group membership for K groups, $T_{i}$ be the time to the indexing event such as death, $t_{ij}$ denote the subject's time of the jth visit since baseline, $y_{ij}$ be the longitudinally observed data at $t_{ij}$. Additionally, let column vectors be defined by listing their elements, for example, $y_{i}=(y_{i1},\cdots \;,y_{im_{i}})$ for $m_{i}$ data points on $t_{i}=(t_{i1},\cdots \;,t_{im_{i}})$. We right‐align the time to the indexing time as $\tau_{ij}=t_{ij}-T_{i}$ and $\tau_{i}=(\tau_{i1},\cdots \;,\tau_{im_{i}})$. The joint distribution of $\left(y_{i},\;T_{i},\;I_{i}\right)$ can be decomposed as a sequence of conditional distributions,

(1) $$\begin{array}{ll} & f\left(y_{i},T_{i},I_{i}|z_{i},\theta \right)\\ & \;=f\left(y_{i}|T_{i},I_{i};z_{i},\theta \right)f\left(T_{i}|I_{i};z_{i},\theta \right)f\left(I_{i}|z_{i},\theta \right)\\ & \;=f\left(y_{i}|T_{i},I_{i};z_{L,i},\theta_{L}\right)f\left(T_{i}|I_{i};z_{S,i},\theta_{S}\right)f\left(I_{i}|z_{I,i},\theta_{I}\right), \end{array}$$

where f(·) is a probability density or probability mass function, $\theta =(\theta_{I},\;\theta_{S},\;\theta_{L})$ is the parameter vector, with components $\theta_{I}$ for the group membership, $\theta_{S}$ for the indexing event time, and $\theta_{L}$ for the longitudinal data sub‐models. Further, let $z_{I,i}$, $z_{S,i}$, and $z_{L,i}$ be subsets of $z_{i}$ for the group membership, the indexing event time, and the longitudinal sub‐models, respectively.

Remark 1 Group memberships may be determined prior to longitudinal data collection, such as categorizing subjects based on baseline smoking status as smokers or nonsmokers. They may also be defined later in the study, as with dementia diagnosis in many aging cohorts. In some cases, group membership for some subjects may remain unknown by the end of the study.

Remark 2 The group‐specific trajectories are right‐aligned to their indexing events, which must be the same type of event to be experienced by all subjects. Our model setup requires that the group membership is determined independently of the indexing event. For example, if death is the indexing event, group memberships may be whether a subject develops a certain condition before death, such as lung cancer or dementia. In our model, the grouping variable indicates whether a condition occurs, not when it occurs. In addition, our framework assumes that, given a sufficiently long follow‐up period, all participants would eventually experience the indexing event. Consequently, the indexing event cannot be the onset of a disease such as dementia or lung cancer in the general population, because not all individuals in general population would ultimately develop the disease.

#### 2.1.2 Models for the Group Membership

The model for the membership indicator $I_{i}$ is defined as

(2) $$f\left(I_{i}=k|z_{I,i},\theta_{I}\right)=p\left(I_{i}=k|z_{I,i},\theta_{I}\right),\;k=1,\cdots \;,K,$$

where $p(I_{i}=k|z_{I,i},\theta_{I})$ is the probability of the ith subject belonging to the kth group given the baseline covariates $z_{I,i}$ and the group membership parameter $\theta_{I}$. These probabilities may be set as fixed values such as the population group proportions or at 1/K for equal group membership proportions. Alternatively, they may be estimated using baseline covariates. Parametric models such as logistic regressions or machine learning models can be adopted. For example, when K=2 for two groups, a logit link can be used as

(3) $$\log\left(\frac{p\left(I_{i}=1|z_{I,i},\theta_{I}\right)}{1-p\left(I_{i}=1|z_{I,i},\theta_{I}\right)}\right)=z_{I,i}^{⊤}\theta_{I}.$$

#### 2.1.3 Models for the Indexing Event Time

AFT models are adopted to model the time to the indexing event [23]. Instead of directly specifying $f(T_{i}|I_{i};z_{S,i},\theta_{S})$, the natural logarithm of the survival time is modeled as an additive model

(4) $$\log\left(T_{i}|I_{i}=k;z_{S,i},\theta_{S}\right)=z_{S,i}^{⊤}\theta_{S,k}+\sigma_{k}e_{i},$$

where $\theta_{S,k}$ is the group‐specific regression parameter vector including an intercept, $\sigma_{k}$ is the variance scale parameter, and $e_{i}$ is the error term. Weibull, gamma, log‐normal, and log‐logistic are some commonly used distributions for AFT models. Denote the survival parameters as the collection $\theta_{S}={\left\{\theta_{S,k},\;\sigma_{k}\right\}}_{k=1}^{K}$.

Remark 3 Although Cox proportional hazards (PH) models are more widely used than AFT models, we adopt AFT models because they are more straightforward to sample from. In contrast, using Cox PH models requires estimation of the baseline hazard function in order to sample survival time from the estimated model [24]. While the nonparametric baseline hazard function in Cox models provides greater flexibility compared to fully parametric approaches, it is generally more difficult to estimate in finite samples and tends to converge more slowly in large sample settings.

#### 2.1.4 Models for the Longitudinal Data

For the longitudinal data $y_{i}$, we assume the distributions $f(y_{i}|T_{i},I_{i};z_{L,i},\theta_{L})$ to be multivariate normal. Let $\theta_{L}=(\phi_{1},\cdots \;,\phi_{K})$, where $\phi_{k}$ is the parameter vector for the kth group, the longitudinal models are defined by the following group‐specific additive models:

(5) $$\left(y_{ij}|T_{i},I_{i}=k;z_{L,i},\phi_{k}\right)=g_{k}\left(\tau_{ij}|T_{i},I_{i}=k;z_{L,i},\phi_{k}\right)+\varepsilon_{kij},$$

with independent and identically distributed error terms distributed as $\varepsilon_{kij}∼N(0,\sigma_{k\varepsilon }^{2})$. The group mean functions $g_{k}(\cdot )$'s are nonlinear functions with a known function form but unknown parameter vectors $\phi_{k}$'s. To account for between‐subject variabilities, mixed effects are adopted for $\phi_{k}$'s. Interested readers are referred to Davidian and Giltinan (1995) [25] for a general treatment of nonlinear mixed effects models.

One particular example of nonlinear mixed effects models is the sigmoidal mixed models [3], which we will use in the simulation and the real data example. The mean function in Equation (5) is modeled as

(6) $$g_{k}\left(\tau_{ij}|T_{i},I_{i}=k;z_{L,i},\phi_{k}\right)=\phi_{ki1}+\frac{\phi_{ki2}-\phi_{ki1}}{1+{\left(\tau_{ij}/\phi_{ki3}\right)}^{\phi_{ki4}}},$$

where the initial value $\phi_{ki1}$, the last level at the indexing event $\phi_{ki2}$, the time of half‐decline $\phi_{ki3}$, and the Hill slope parameter $\phi_{ki4}$ may depend on the covariate vector $z_{L,i}$ as follows

(7) $$\begin{array}{ll} \phi_{ki1} & =\psi_{ki1}+\alpha_{ki1}=z_{L,ki1}^{⊤}\beta_{k1}+\alpha_{ki1}, \end{array}$$

(8) $$\begin{array}{ll} \phi_{ki2} & =\psi_{ki2}+\alpha_{ki2}=z_{L,ki2}^{⊤}\beta_{k2}+\alpha_{ki2}, \end{array}$$

(9) $$\begin{array}{ll} \phi_{ki3} & =\psi_{ki3}=z_{L,ki3}^{⊤}\beta_{k3}, \end{array}$$

(10) $$\begin{array}{ll} \phi_{ki4} & =\psi_{ki4}=z_{L,ki4}^{⊤}\beta_{k4}, \end{array}$$

where $z_{L,ki1}$, $z_{L,ki2}$, $z_{L,ki3}$ and $z_{L,ki4}$ are subsets of $z_{L,i}$ which can reduce to a column of ones for an intercept only. Subject‐specific random effects are mutually independently distributed as $\alpha_{ki1}∼N(0,\sigma_{k1}^{2})$ and $\alpha_{ki2}∼N(0,\sigma_{k2}^{2})$. Parameter vector $\phi_{k}$ is the collection of these group‐specific parameters as $\phi_{k}=(\beta_{k1},\;\beta_{k2},\;\beta_{k3},\;\beta_{k4},\;\sigma_{k1}^{2},\;\sigma_{k2}^{2})$.

Consequently, the longitudinal data vector $y_{i}$ conditional on $\left(T_{i},I_{i}=k;z_{L,i},\phi_{k}\right)$ follow a multivariate normal distribution

(11) $$\begin{array}{ll} y_{i}|T_{i},I_{i}=k;z_{L,i},\phi_{k} & ∼f_{k}\left(y_{i}|T_{i},I_{i}=k;z_{L,i},\phi_{k}\right) \end{array}$$

(12) $$\begin{array}{ll} & ∼N\left(\mu_{ki},\sum_{ki}\right), \end{array}$$

(13) $$\begin{array}{ll} \mu_{ki} & =E\left\{g_{k}\left(\tau_{i}|T_{i},I_{i}=k;z_{L,i},\phi_{k}\right)\right\}, \end{array}$$

(14) $$\begin{array}{ll} \sum_{ki} & =A_{ki}B_{k}A_{ki}^{⊤}+\sigma_{k\varepsilon }^{2}I_{m_{i}}, \end{array}$$

(15) $$\begin{array}{ll} A_{ki} & ={\left(\begin{array}{ccc} \frac{{\left(\tau_{i1}/\psi_{k3i}\right)}^{\psi_{k4i}}}{1+{\left(\tau_{i1}/\psi_{k3i}\right)}^{\psi_{k4i}}} & \cdots & \frac{{\left(\tau_{im_{i}}/\psi_{k3i}\right)}^{\psi_{k4i}}}{1+{\left(\tau_{im_{i}}/\psi_{k3i}\right)}^{\psi_{k4i}}}\\ \frac{1}{1+{\left(\tau_{i1}/\psi_{k3i}\right)}^{\psi_{k4i}}} & \cdots & \frac{1}{1+{\left(\tau_{im_{i}}/\psi_{k3i}\right)}^{\psi_{k4i}}} \end{array}\right)}^{⊤}, \end{array}$$

(16) $$\begin{array}{ll} B_{k} & =\text{diagonal}\left\{\sigma_{k1}^{2},\;\sigma_{k2}^{2}\right\}. \end{array}$$

Remark 4 Following Capuano and Wagner's [3] approach, we also only allow fixed effects in $\phi_{ki3}$ and $\phi_{ki4}$. When the time to the indexing event $T_{i}$ has not been observed, the mapped time $\tau_{ij}$'s are consequently random variables. Modeling either $\phi_{ki3}$ or $\phi_{ki4}$ as mixed effects will lead to two random variables with nonlinear relationships in the denominator in Equation (6). This will not only make the integrations in the next section analytically intractable, but also make the Monte Carlo approximations more challenging.

Remark 5 Random effects $\alpha_{ki1}$ and $\alpha_{ki2}$ are assumed independent. This represents a simplification of the more general case where the two random effects may be correlated, as in Capuano and Wagner [3]. We adopt this independence to ameliorate potential identifiability issues, given that the overall modeling framework is already more complex than traditional models that consider only longitudinal outcomes.

Remark 6 The Hill slope parameter, $\phi_{ki4}$, quantifies the steepness of the sigmoidal functions. Larger Hill slope values indicate more rapid changes, whereas smaller values indicate more gradual changes over time. It is of practical interest to investigate whether the Hill slopes are associated with modifiable factors, such as education, alcohol drinking, smoking, and body mass index (BMI), and subsequently, how to modify these factors to decelerate deterioration or accelerate recoveries along time.

### 2.2 Parameter Estimation and Inference

#### 2.2.1 Parameter Estimation

Denote Y as the observed data, $S_{I,T,y}$ the subset of subjects with fully observed data, $S_{I,y}$ the subset with observed group memberships and longitudinal data but not the indexing events, $S_{T,y}$ the subset with observed indexing events and longitudinal data but not the group memberships, and $S_{y}$ the subset with observed longitudinal data but not the group memberships nor the indexing events. The likelihood function for the parameter vector $\theta =(\theta_{I},\;\theta_{S},\;\theta_{L})$ is

(17) $$\begin{array}{ll} L\left(\theta |Y\right) & =\underset{i\in S_{I,T,y}}{\prod }f\left(y_{i},T_{i},I_{i}|z_{i},\theta \right)\\ & \;\times \underset{i\in S_{I,y}}{\prod }\int_{T_{i}}f\left(y_{i}|T_{i},I_{i};z_{L,i},\theta_{L}\right)\\ & \;\times f\left(T_{i}|I_{i};z_{S,i},\theta_{S}\right)f\left(I_{i}|z_{I,i},\theta_{I}\right)dT_{i}\\ & \;\times \underset{i\in S_{T,y}}{\prod }\left\{\overset{K}{\underset{i=1}{\sum }}f\left(y_{i}|T_{i},I_{i};z_{L,i},\theta_{L}\right)\right.\\ & \;\times \left.f\left(T_{i}|I_{i};z_{S,i},\theta_{S}\right)f\left(I_{i}|z_{I,i},\theta_{I}\right)\right\}\\ & \;\times \underset{i\in S_{y}}{\prod }\left\{\overset{K}{\underset{i=1}{\sum }}\int_{T_{i}}f\left(y_{i}|T_{i},I_{i};z_{L,i},\theta_{L}\right)\right.\\ & \;\times \left.f\left(T_{i}|I_{i};z_{S,i},\theta_{S}\right)f\left(I_{i}|z_{I,i},\theta_{I}\right)dT_{i}\right\}. \end{array}$$

The simplest situation is that all the subjects have fully observed data. The likelihood can be decomposed as

(18) $$\begin{array}{ll} L\left(\theta |Y\right) & =\underset{i}{\prod }f\left(y_{i},T_{i},I_{i}|z_{i},\theta \right)\\ & =\left\{\underset{i}{\prod }f\left(y_{i}|T_{i},I_{i};z_{L,i},\theta_{L}\right)\right\}\\ & \;\times \left\{\underset{i}{\prod }f\left(T_{i}|I_{i};z_{S,i},\theta_{S}\right)\right\}\\ & \;\times \left\{\underset{i}{\prod }f\left(I_{i}|z_{I,i},\theta_{I}\right)\right\}. \end{array}$$

If we further assume that $\theta_{I}$, $\theta_{S}$, and $\theta_{L}$ have no shared components, the three sub‐models can be fitted separately by maximizing each one's likelihood. In this situation, the logistic model likelihood can be maximized using standard numeric methods such as the Fisher scoring method as in SAS PROC LOGISTIC (SAS Institute, Cary, NC, USA). The survival parameter $\theta_{l}$ can be estimated using standard software for AFT models such as SAS PROC LIFEREG using the ridge‐stabilized Newton‐Raphson algorithm. The longitudinal model likelihood can be maximized by adaptive Gaussian quadrature as in SAS PROC NLMIXED.

In the more general cases where the group memberships and the indexing events are not both completely observed and $\theta_{I}$, $\theta_{S}$, and $\theta_{L}$ may have shared components, expectation maximization (EM) algorithms can be adopted to maximize the joint likelihood. Let $y=(y_{1},\cdots \;,y_{n})$ be the longitudinal data from all the n subjects, $\alpha =(\alpha_{1},\cdots \;,\alpha_{n})$ be the random effects of the longitudinal models, $T=(T_{1},\cdots \;,T_{n})$ the indexing event time, $I=(I_{1},\cdots \;,I_{n})$ be the group memberships, $z=(z_{1},\cdots \;,z_{n})$ be the covariates, and $\theta^{\prime }$ the working parameter values, the Q function is

(19) $$\begin{array}{ll} Q\left(\theta |\theta^{\prime }\right) & =\int \log\left\{f\left(y,\alpha ,T,I|z,\theta \right)\right\}\\ & \;f\left(\alpha ,T,I|y;z,\theta^{\prime }\right)d\alpha \;dT\;dI. \end{array}$$

Since the Q function is analytically intractable in general for the proposed models, we adopt the SAEM algorithms to maximize the likelihood [21, 22]. Briefly, the Q function in the E step is approximated by m(s) random draws at step s from $f\left(\alpha ,T,I|y,z,\theta^{\left(s\right)}\right)$ as

(20) $${\hat{Q}}_{s}=\left(1-w_{s}\right){\hat{Q}}_{s-1}+w_{s}\overset{m(s)}{\underset{l=1}{\sum }}\log\left\{f\left(y,\alpha^{\left(l\right)},T^{\left(l\right)},I^{\left(l\right)}|z,\theta \right)\right\},$$

where $w_{s}$'s are step sizes satisfying (i) $0\leq w_{s}\leq 1$, (ii) $\sum_{s=1}^{\infty }w_{s}=\infty$, and (iii) $\sum_{s=1}^{\infty }w_{s}^{2}<\infty$. The joint draws of ${\left\{\alpha^{\left(l\right)},T^{\left(l\right)},I^{\left(l\right)}\right\}}_{l=1}^{m(s)}$ can be obtained sequently as follows.

a. Draw ${\left\{I^{\left(l\right)}\right\}}_{l=1}^{m(s)}$ from $f\left(I|y;z,\theta^{\left(s\right)}\right)$.
b. For each $I^{\left(l\right)}$, draw $T^{\left(l\right)}$ from $f\left(T|I^{\left(l\right)},y;z,\theta^{\left(s\right)}\right)$.
c. For each $\left(T^{\left(l\right)},I^{\left(l\right)}\right)$, draw $\alpha^{\left(l\right)}$ from $f\left(\alpha^{\left(l\right)}|T,I^{\left(l\right)},y;z,\theta^{\left(s\right)}\right)$.

After the SAEM algorithms converge, in addition to the maximum likelihood estimates (MLEs) $\hat{\theta }$, their asymptotic variances are also available by using Louis formula [26].

Remark 7 For model identifiability considerations, we require that a subset of subjects should have both indexing event time and group memberships available, though potential relaxations are possible.

#### 2.2.2 Hypothesis Testing, Confidence Intervals, and Model Selection

Hypothesis testing can be performed based on the likelihood estimates using the Wald test, score test, or likelihood ratio test. Wald‐type confidence intervals can be constructed using MLEs and their asymptotic variances. Model selections for nested models can be performed by testing the corresponding parameters, while for non‐nested models by information criteria such as AIC.

### 2.3 Applications of the Proposed Method

#### 2.3.1 Group Membership Classification

In addition to estimating the group‐specific trajectories and studying covariate effects on these trajectories, another application of the proposed method is the classification of group membership based on the longitudinal trajectories for subjects whose group memberships are unknown. For subjects with data on the longitudinal profiles and the time to the indexing events but not their group memberships, their group membership probabilities can be straightforwardly updated using Bayes' rule. We will refer to these subjects as target subjects hereafter. For a target subject, let $z_{i^{\prime }}$ be the baseline covariate vector, $T_{i^{\prime }}$ be the time to the indexing event, and $y_{i^{\prime }}$ be the longitudinal data vector from this target subject with the original time vector $t_{i^{\prime }}=(t_{i^{\prime }1},\cdots \;,t_{i^{\prime }m_{i^{\prime }}})$ and the transformed time vector $\tau_{i^{\prime }}=t_{i^{\prime }}-T_{i^{\prime }}$. The probability of the target subject belonging to the kth group is

(21) $$\begin{array}{cc} & p\left(I_{i^{\prime }}=k|y_{i^{\prime }}\right)\\ & \;=\frac{f\left(I_{i^{\prime }}=k|z_{I,i^{\prime }},{\hat{\theta }}_{I}\right)f\left(y_{i^{\prime }}|I_{i^{\prime }}=k;z_{L,i^{\prime }},{\hat{\theta }}_{L}\right)}{\sum_{l=1}^{K}f\left(I_{i^{\prime }}=l|z_{I,i^{\prime }},{\hat{\theta }}_{I}\right)f\left(y_{i^{\prime }}|I_{i^{\prime }}=l;z_{L,i^{\prime }},{\hat{\theta }}_{L}\right)}, \end{array}$$

where ${\hat{\theta }}_{I}$ and ${\hat{\theta }}_{L}$ are the MLE of the parameter vectors.

Remark 8 One such situation is that the group membership identifications are either expensive or destructive or both, such that only a limited number of subjects have complete data on the longitudinal profiles, the time to the indexing events, and the group memberships.

#### 2.3.2 Group Membership Predictions

A more complex application of the proposed models is to predict the group membership of a subject whose indexing event has not occurred, for example, whether an alive subject with normal cognitive functions will develop dementia before death. Suppose we have groups of subjects whose memberships and indexing events are both observed, based on which the group‐specific trajectories can be estimated. For the target subject, let $z_{i^{\prime }}$ be the baseline covariate vector and $y_{i^{\prime }}$ be the longitudinal data vector from this target subject with the original time vector $t_{i^{\prime }}=(t_{i^{\prime }1},\cdots \;,t_{i^{\prime }m_{i^{\prime }}})$ and the transformed time vector $\tau_{i^{\prime }}=t_{i^{\prime }}-T_{i^{\prime }}$. The group memberships will be predicted by comparing the alignments of $y_{i^{\prime }}$ to the group‐specific trajectories and combining this information with the prior probabilities before considering the longitudinal data.

The likelihood of the target subject conditional on belonging to the kth group is

(22) $$\begin{array}{cc} & f\left(y_{i^{\prime }}|I_{i^{\prime }}=k;z_{L,i^{\prime }},z_{S,i^{\prime }},\theta_{L},\theta_{S}\right)\\ & \;=\int f\left(y_{i^{\prime }}|I_{i^{\prime }}=k,T_{i^{\prime }};z_{L,i^{\prime }},\theta_{L}\right)\\ & \;f\left(T_{i^{\prime }}|I_{i^{\prime }}=k;z_{S,i^{\prime }},\theta_{S}\right)dT_{i^{\prime }}, \end{array}$$

which is defined on the transformed time domain $\tau_{i^{\prime }}$ as detailed in Section 2.1.4. The group membership predictions are then calculated using Equation (21). The integrations in Equation (22) are analytically intractable, which we adopt Monte Carlo integration to approximate by drawing indexing event time from the estimated survival distributions indexed by ${\hat{\theta }}_{S}$ and plugging in the estimated longitudinal parameters ${\hat{\theta }}_{L}$, as listed in the follows.

a. Draw indexing event time $\left\{T_{ki^{\prime }}^{\left(r\right)}|I_{i^{\prime }}=k\right\}$ using $z_{S,i^{\prime }}$ and MLE ${\hat{\theta }}_{S}$ for k=1,⋯,K. Keep R copies that are bigger than the censor time.
b. For each of indexing event time draw,
  b.1. first calculate $\tau_{ki^{\prime }}^{\left(r\right)}=\tau_{i^{\prime }}-T_{ki^{\prime }}^{\left(r\right)}$,
  b.2. then calculate $f^{\left(r\right)}(y_{i^{\prime }}|I_{i^{\prime }}=k,T_{ki^{\prime }}^{\left(r\right)};z_{L,i^{\prime }},{\hat{\theta }}_{L})$ for k=1,⋯,K.
c. Average the R copies to obtain
(23) $$\begin{array}{cc} & \tilde{f}\left(y_{i^{\prime }}|I_{i^{\prime }}=k;z_{L,i^{\prime }},z_{S,i^{\prime }},{\hat{\theta }}_{L},{\hat{\theta }}_{S}\right)\\ & \;=\frac{1}{R}\overset{R}{\underset{r=1}{\sum }}f^{\left(r\right)}(y_{i^{\prime }}|I_{i^{\prime }}=k,T_{ki^{\prime }}^{\left(r\right)};z_{L,i^{\prime }},{\hat{\theta }}_{L}). \end{array}$$

The estimated marginal likelihood $\tilde{f}(y_{i^{\prime }}|I_{i^{\prime }}=k;z_{L,i^{\prime }},z_{S,i^{\prime }},{\hat{\theta }}_{L},{\hat{\theta }}_{S})$ for k=1,⋯,K are then plugged into Equation (21) to calculate the prediction probabilities.

#### 2.3.3 Modifications of Indexing Event Time Predictions

Another application is to use the longitudinal profiles to modify the indexing event time predictions. Consider the situations where the group memberships are known at the beginning stage of the longitudinal data and we have groups of subjects with complete data. Consequently, there is no need to model the group membership probabilities, and the AFT models and the nonlinear mixed effects models can be estimated from the observed data. Suppose that we have a target subject with longitudinal data whose indexing event has not occurred. Similarly, let the baseline covariate vector be $z_{i^{\prime }}$ and the longitudinal data be $y_{i^{\prime }}$. Before considering the longitudinal profiles, the indexing event time $T_{ki^{\prime }}$ is distributed as $f(T_{ki^{\prime }}|I_{i^{\prime }}=k;z_{S,i^{\prime }},{\hat{\theta }}_{S})$ according to

(24) $$\log\left(T_{ki^{\prime }}|I_{i^{\prime }}=k;z_{S,i^{\prime }},{\hat{\theta }}_{S}\right)=z_{S,i^{\prime }}^{⊤}{\hat{\theta }}_{S,k}+{\hat{\sigma }}_{k}e_{i^{\prime }},$$

by plugging in the MLE ${\hat{\theta }}_{S}$. The indexing event time prediction is based on this distribution, which can be further updated by including the information in the longitudinal data as

(25) $$\begin{array}{cc} & f\left(T_{ki^{\prime }}|y_{i^{\prime }},I_{i^{\prime }}=k;z_{S,i^{\prime }},z_{L,i^{\prime }},{\hat{\theta }}_{S},{\hat{\theta }}_{L}\right)\\ & \;=\frac{f\left(y_{i^{\prime }}|T_{ki^{\prime }},I_{i^{\prime }}=k;z_{L,i^{\prime }},{\hat{\theta }}_{L}\right)f\left(T_{ki^{\prime }}|I_{i^{\prime }}=k;z_{S,i^{\prime }},{\hat{\theta }}_{S}\right)}{f\left(y_{i^{\prime }}|I_{i^{\prime }}=k;z_{L,i^{\prime }},z_{S,i^{\prime }},{\hat{\theta }}_{L},{\hat{\theta }}_{S}\right)}. \end{array}$$

The evaluation of $f(y_{i^{\prime }}|I_{i^{\prime }}=k;z_{L,i^{\prime }},z_{S,i^{\prime }},{\hat{\theta }}_{L},{\hat{\theta }}_{S})$ needs integration over $T_{ki^{\prime }}$, which is analytically intractable. Instead, the distribution in Equation (25) can be approximated by reweighting Monte Carlo draws as the follows.

a. Draw the indexing event time $\left\{T_{ki^{\prime }}^{\left(r\right)}|I_{i^{\prime }}=k\right\}$ using $z_{S,i^{\prime }}$ and MLE ${\hat{\theta }}_{S}$. Keep R copies that are bigger than the censor time.
b. For each event time draw, calculate the raw weight as
$$v_{i^{\prime }}^{\left(r\right)}=f^{\left(r\right)}\left(y_{i^{\prime }}|I_{i^{\prime }}=k;T_{ki^{\prime }}^{\left(r\right)},z_{L,i^{\prime }},{\hat{\theta }}_{L}\right).$$
c. Normalize the weight as
$$w_{i^{\prime }}^{\left(r\right)}=\frac{v_{i^{\prime }}^{\left(r\right)}}{\sum_{l=1}^{R}v_{i^{\prime }}^{\left(l\right)}}.$$
d. The weighted sample ${\left\{(T_{ki^{\prime }}^{\left(r\right)},w_{i^{\prime }}^{\left(r\right)})\right\}}_{r=1}^{R}$ is an approximation to $f(T_{ki^{\prime }}|y_{i^{\prime }},I_{i^{\prime }}=k;z_{S,i^{\prime }},z_{L,i^{\prime }},{\hat{\theta }}_{S},{\hat{\theta }}_{L})$.

## 3 Simulation

### 3.1 General Setup

Simulation studies are conducted to evaluate the finite sample performance of the proposed method in situations similar to the real data example in Figure 1. The survival and longitudinal parameters are selected to mimic the real data example with some variations, especially that the group proportions are set at 50%. Weibull distribution is adopted for the survival models with the mean in Equation (4), which is also the natural logarithm of the scale parameter, as

(26) $$\begin{array}{ll} z_{S,i}^{⊤}\theta_{S,k} & =3.9642-0.0249\times \left(\text{baseline age}-75\right)\\ & \;+0.1700\times I\left\{\text{if female}\right\}+0.0596\times I\left\{\text{if dementia}\right\}\\ & \;+0.1784\times I\left\{\text{if overweight}\right\}+0.0074\times \text{years of education}\\ & \;-0.0460\times I\left\{\text{if smoking}\right\}-0.1363\times I\left\{\text{if depressed}\right\}\\ & \;-0.0395\times I\left\{\text{if hypertension}\right\}-0.1042\times I\left\{\text{if ever stroke}\right\}\\ & \;-0.1052\times I\left\{\text{if diabetic}\right\}, \end{array}$$

where I{·} is the indicator function. Baseline age is defined as the age at enrollment and is generated from Uniform[65,85]. Female, dementia, overweight, smoking, depression,hypertension, stroke, and diabetes are generated as Bernoulli random variables with probabilities 0.50, 0.50, 0.60, 0.58, 0.12, 0.70, 0.16, and 0.26, respectively. Years of education is generated as a discrete random number on integers 0–16 with probabilities 0.0017, 0.0023, 0.0035, 0.0250, 0.0285, 0.0163, 0.0314, 0.0483, 0.1651, 0.0895, 0.1029, 0.1151, 0.2070, 0.0849, 0.0250, 0.0343, and 0.0192, respectively. For the shape parameter, which is the reciprocal of the variance scale parameter $\sigma_{k}$, both $1/\sigma_{k}=3$ for larger variance and $1/\sigma_{k}=10$ for smaller variances are adopted.

For the longitudinal parameters in Equation (6), let, $\phi_{ki1}=\psi_{k1}+\alpha_{k1i}$ with $\psi_{k1}=90$ and $\alpha_{k1i}∼N(0,\sigma_{k1}^{2}=2)$ for both k=1 and k=2 for the initial values. The last levels at death are $\phi_{1i2}=\psi_{12}+\alpha_{12i}$ with $\psi_{12}=30$ and $\alpha_{12i}∼N(0,\sigma_{12}^{2}=90)$ for k=1 the dementia group and $\phi_{2i2}=\psi_{22}+\alpha_{22i}$ with $\psi_{22}=50$ and $\alpha_{22i}∼N(0,\sigma_{22}^{2}=90)$ for k=2 the normal group. The times of half decline are $\phi_{13}=\phi_{23}=-8$ years. The Hill slope parameters are modeled both as with and without the effects of years of education. When there are no years of education effects, we have $\phi_{14}=0.8$ and $\phi_{24}=0.3$. When there are years of education effects, the effects are modeled as −0.014×(years of education−9), indicating slower decline for more education years. The maximal difference in the Hill slope parameters is 0.2240 between a person with zero year of education and another with 16 years of education, which we consider as a moderate effect size. The differences of the parameters between the dementia group and the normal group are in the last levels and the Hill slope parameters, such that the dementia group declines at a faster rate to a lower level.

### 3.2 Parameter Estimate

We first evaluate the longitudinal parameter estimate using Matlab function nlmefitsa for Group 1 without the education effects, that is, $\psi_{11}=90$, $\sigma_{11}^{2}=2$, $\psi_{12}=30$, $\sigma_{12}^{2}=90$, $\phi_{1i3}=-8$, and $\phi_{1i4}=0.8$. By assuming known group memberships and survival time, we generate 10 observations per subject over time [−10,0] for N=100, 200 and 500 subjects with error variance $\sigma_{\varepsilon }^{2}=25$. The biases and coverage probabilities of 95% confidence intervals from 100 replicates are displayed in Table 1. Overall, the biases are small which suggests that the longitudinal parameters can be reliably estimated when N=100 per group, though more subjects may be needed to estimate the variance parameters. The coverage probabilities are less than optimal, suggesting that the corresponding standard error estimates from Matlab function nlmefitsa may need to be adjusted for better coverage.

**TABLE 1:** Biases and coverage probabilities of 95% confidence intervals for Group 1 longitudinal parameters.

	Bias			Coverage probability		
Parameter	N=100	N=200	N=500	N=100	N=200	N=500
$\psi_{11}=90$	2.73	3.33	3.17	1.00	0.99	0.86
$\psi_{12}=30$	−1.58	−1.59	−1.41	0.92	0.88	0.65
$\phi_{13}=-8$	−0.48	−0.64	−0.63	0.98	0.98	0.98
$\phi_{14}=0.8$	−0.06	−0.06	−0.06	0.87	0.80	0.52
$\sigma_{11}^{2}=2$	−1.11	−0.15	−0.07			
$\sigma_{12}^{2}=90$	−3.25	0.10	−1.83			

We further evaluate the convergence and biases of the full SAEM approach with N=100 subjects per group having completed data. Another N=100 subjects per group with both missing group memberships and missing indexing event time are generated by introducing uniform missing after Year 3 of follow up. The error variances of the longitudinal data are set to $\sigma_{\varepsilon }^{2}=5$ and half decline time $\phi_{13}=\phi_{23}=-4$ years. The full SAEM algorithm is initialized using parameter estimates from the subjects with complete data. Following Kuhn and Lavielle 2005's suggestion [22], we let the step sizes be $w_{s}=1$ for s=1,⋯,100 and $w_{s}=1/(s-99)$ for s>100. Figure 2 shows the trace plots of selected parameters from one simulation, which suggest that the survival parameter estimates converge after a few SAEM iterations, while the longitudinal parameter estimates require more SAEM iterations to converge. The biases from 100 replicates are summarized in Table 2. It shows that the survival parameter estimates have small biases. The longitudinal parameter estimates are in general more biased than those in Table 1, even with more longitudinal data included. The coverage probabilities are all below 25% hence not displayed.

**FIGURE 2:**
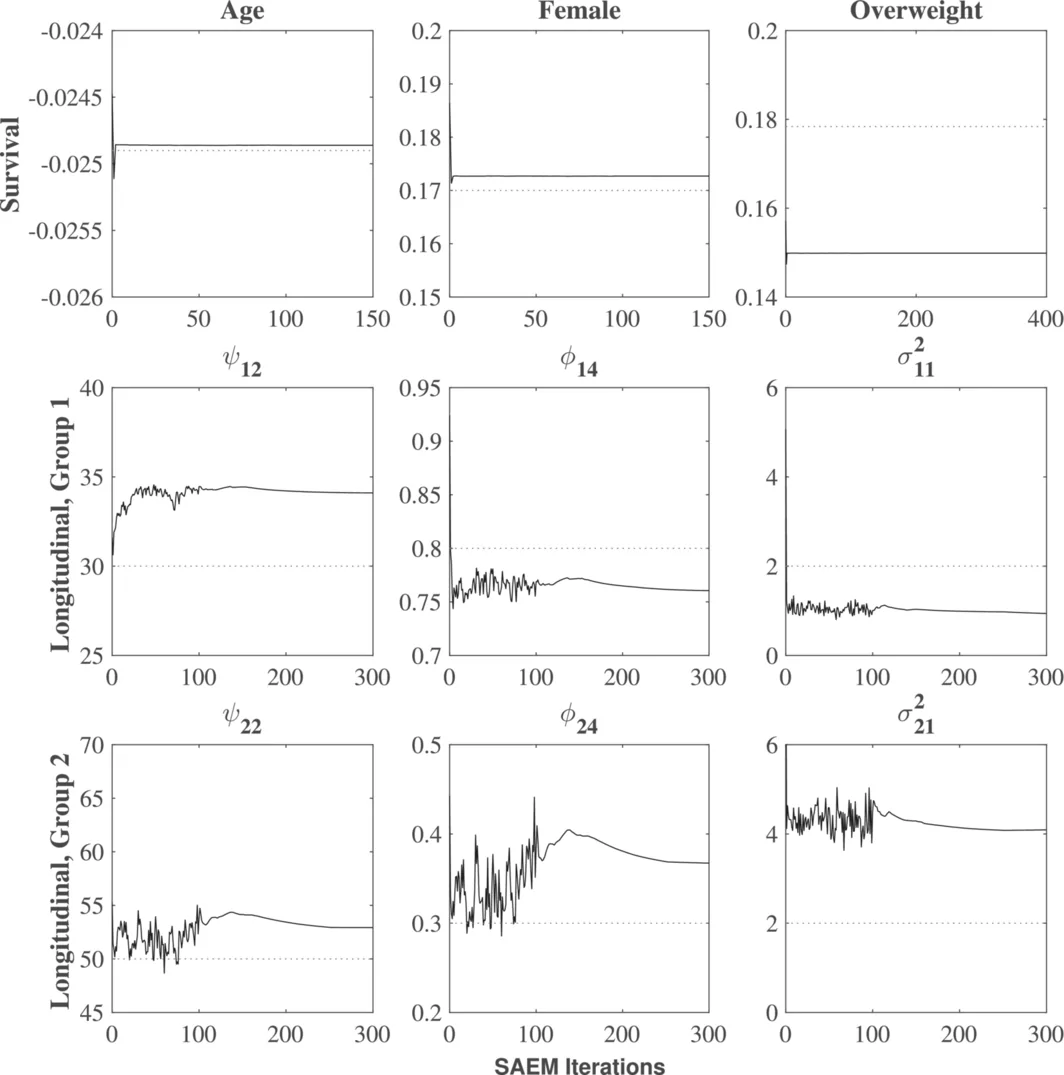
Simulation: Trace plots of selected parameters in a full SAEM algorithm. In each sub‐figure, the solid line is the trace plot of the parameter estimate, while the dotted line is the true parameter value.

**TABLE 2:** Biases of a full SAEM algorithm with missing group memberships and survival time.

Survival parameters				Longitudinal parameters		
Parameter	True value	Bias	Coverage	Parameter	True value	Bias
				Group 1		
Intercept	3.9642	−0.0011	0.78	$\psi_{11}$	90	−6.3083
Dementia	0.0596	−0.0121	0.78	$\psi_{12}$	30	2.8525
Age	−0.0249	0.0003	0.76	$\phi_{13}$	−4	0.5015
Female	0.1700	−0.0002	0.75	$\phi_{14}$	0.8	0.1781
Overweight	0.1784	−0.0050	0.74	$\sigma_{11}^{2}$	2	0.5775
Years of Education	0.0074	−0.0014	0.65	$\sigma_{12}^{2}$	90	2.5719
Smoking	−0.0460	0.0102	0.64	Group 2		
Depression	−0.1363	0.0091	0.78	$\psi_{21}$	90	−9.1730
Hypertension	−0.0395	0.0037	0.69	$\psi_{22}$	50	9.3258
Stroke	−0.1042	0.0066	0.75	$\phi_{23}$	−4	0.0315
Diabetes	−0.1052	0.0110	0.75	$\phi_{24}$	0.3	0.2805
Weibull shape	10	0.6429	0.64	$\sigma_{21}^{2}$	2	9.8054
				$\sigma_{22}^{2}$	90	−18.5923

Previous studies of nonlinear mixed effects models have shown that standard errors based on the asymptotic Fisher information matrix can underestimate the finite‐sample uncertainty of parameter estimates, particularly for variance components and when the data are relatively far from the asymptotic regime [27, 28]. These findings provide an important context for the less‐than‐nominal coverage observed in our simulations and suggest that the conventional asymptotic standard errors associated with the SAEM estimates may not fully reflect finite‐sample uncertainty in some settings. Bootstrap‐based inference is therefore a potentially useful alternative for obtaining more robust measures of parameter uncertainty [28, 29]. Because the objective of our subsequent analyses is prediction rather than inference on the population parameters themselves, we did not undertake an additional set of bootstrap simulations in this study.

In the following simulations of group membership predictions and modifications of indexing event time predictions and in the real data example in the next section, we only use the complete data to estimate the parameters separately for the three sub‐models. This is to mimic a common practice where complete data have been observed from a collection of subjects, while the predictions of either group membership or time‐to‐indexing‐event are needed for subjects that are still under study. Additionally, including target subjects of prediction in parameter estimation may lead to model overfitting to the target subjects.

### 3.3 Group Membership Predictions

For each combination of Weibull shape parameters 3 and 10 and with or without effects of years of education, we generate N=100,200,500 subjects per group for training, and N=1000 target subjects per group for dementia prediction; hence there are 2×2×3=12 scenarios. The simulation data are then generated according to the following steps.

1. The survival time $T_{i}$ is generated according to the Weibull distributions in the unit of years for each subject.
2. With a generated survival time, the observation time vector $t_{i}$ is generated as annual visits with random deviations by Uniform[−0.1,0.1] years. The mapped time vector is thus $\tau_{i}=t_{i}-T_{i}$.
3. The mean vector of the longitudinal data is generated according the longitudinal parameters.
4. The longitudinal data are generated by adding noises to the mean vectors with independent normal noises from $\varepsilon_{ij}∼N(0,\sigma_{\varepsilon }^{2})$ with five different variation levels as $\sigma_{\varepsilon }^{2}=1,5,10,25,50$.
5. The target subjects' data are censored uniformly on $\left[0,T_{i}\right]$.

For each of these 12×5=60 settings, the training data are used to estimate the survival parameters with the correct models. The training data are then used to estimate the longitudinal parameters with both the correct and wrong models with respect to the effects of years of education. With the estimated survival and longitudinal parameters, the prediction probabilities are calculated for the target subjects with R=1000 survival time draws and prior group membership probabilities $p(I_{i}=1|z_{i},\theta )=p(I_{i}=2|z_{i},\theta )=0.5$. Consequently, there are 60×2=120 sets of prediction probabilities.

Figure 3 displays the boxplots overlaid with swarm charts of the prediction probabilities of one setting with Weibull shape parameter 10, correct longitudinal model without the effects of years of education, N=500 per group training subjects, and longitudinal error variance $\sigma_{\varepsilon }^{2}=25$. The figure shows that more than 50% of the subjects have correct prediction probabilities >0.90 and more than three‐quarters subjects have correct prediction probabilities >0.60. Under the receiver operating characteristic (ROC) analysis, the area under the ROC curve (AUC) is 0.9397.

**FIGURE 3:**
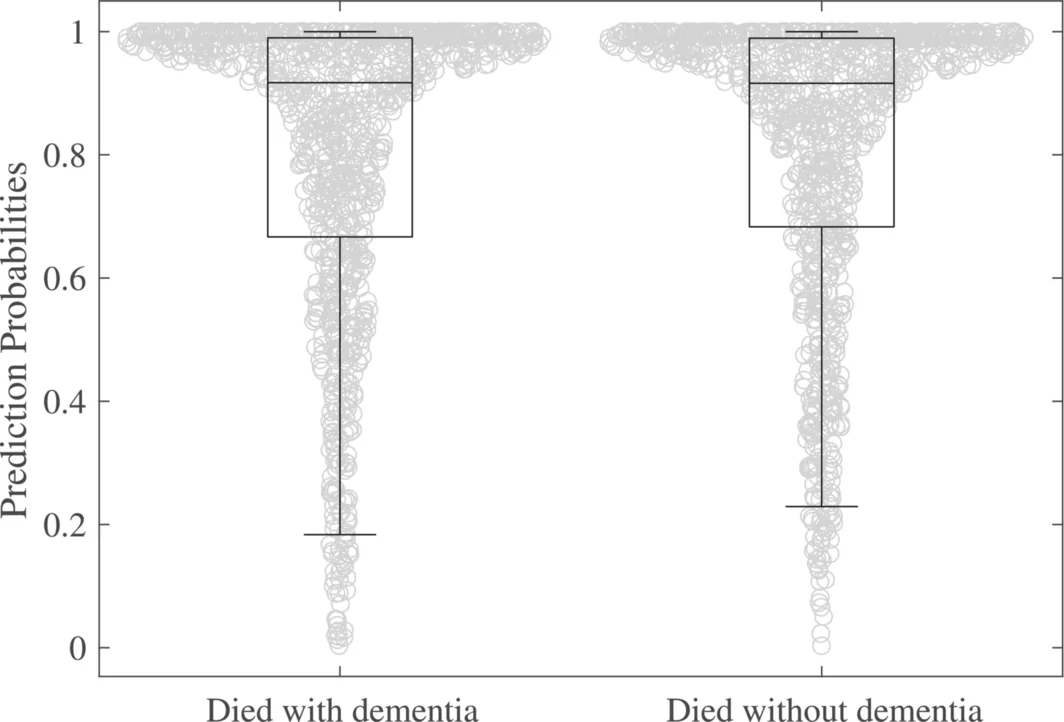
Simulation: Prediction probabilities of belonging to the true groups for Weibull shape parameter 10, correct longitudinal model without the effects of years of education, N=500 per group training subjects, and longitudinal error variance $\sigma_{\varepsilon }^{2}=25$.

The AUC values from the 120 sets are displayed in Figure 4. Overall, the proposed method works well in this simulation setting with all the AUCs >0.85. Under the same setting except the longitudinal error variances, AUCs are larger for smaller variances. Under the same setting except the Weibull shape parameters, AUCs are larger for shape parameter 10 than for 3. The AUCs under N=200 are higher than AUCs under N=100 but the differences between N=200 and N=500 are minimal, suggesting that N=200 per group is already sufficient for this simulation setting. The model performance is also robust against model misspecifications under moderate effect sizes on the Hill slope parameters, as showed in the right column of Figure 4.

**FIGURE 4:**
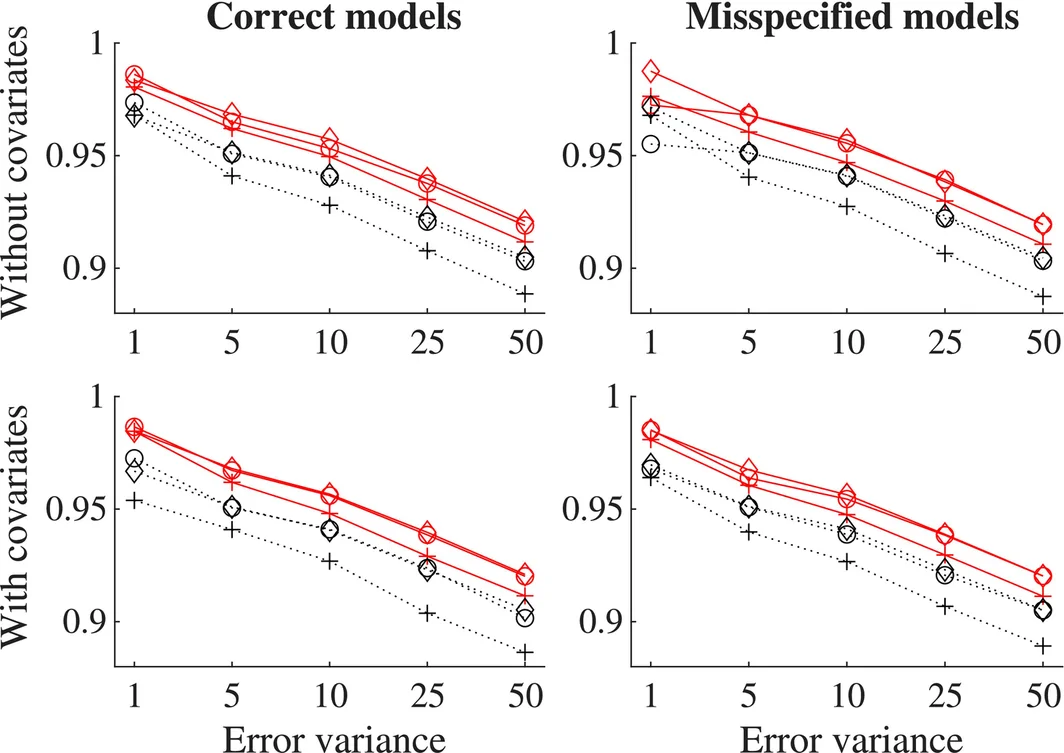
Simulation: AUC values. The red solid lines are for Weibull shape parameter 10 while the black dotted lines for Weibull shape parameter 3. Lines with plus signs are for N=100 training subjects per group, with circles for N=200, and with diamonds for N=500. The upper row is for the true models without and the lower row for the true models with the effects of years of education. The left column is for the working models being correct while the right column for the working models being misspecified with respect to whether the effects of years of education are included on the Hill slope parameters.

Remark 9 The estimated parameters and random effects can be used to predict future longitudinal trajectories. We calculated the mean squared prediction error (MSPE) and the median absolute prediction error (MAPE) for each of the 1000 subjects from their censored time to the last time points for the same setting as in Figure 3 with Weibull shape parameter 10, N=500 per group training subjects, no effects of years of education, and longitudinal error $\sigma_{\epsilon }^{2}$ = 25. The medians (1st, 3rd quartiles) of these prediction errors are 16.19 (3.46, 55.85) for MSPE and 3.78 (1.77, 6.99) for MAPE for the group with dementia, and 12.74 (2.52, 61.64) for MSPE and 3.47 (1.51, 7.56) for MAPE for the group without dementia.

Another simulation is performed where the data generating functions are different from Equation (6) while the working longitudinal model is still Equation (6). Same settings as previous are used, except that the following three functions are adopted to generate the longitudinal data.

1. The reverse Gompertz function
$$g_{k}(\tau_{ij}|T_{i},I_{i}=k;\phi_{i})=a_{ki}\exp\left\{-b_{ki}\exp\left(-c_{ki}\tau_{ij}\right)\right\}+d_{k},$$
  where $a_{1i}∼N(60,2)$, $a_{2i}∼N(40,2)$, $b_{1i}∼\text{Uniform}[10,20]$, $b_{2i}∼\text{Uniform}[0.15,0.35]$, $c_{1i}∼\text{Uniform}[-0.6,-0.4]$, $c_{2i}∼\text{Uniform}[-0.6,-0.4]$, $d_{1}$ = 30 and $d_{2}$ = 50.
2. The hyperbolic tangent function
$$g_{k}(\tau_{ij}|T_{i},I_{i}=k;\phi_{i})=-(a_{ki}-b_{ki})\tanh(c_{k}\tau_{ij})+b_{ki},$$
  where the hyperbolic tangent function tanh(s)={exp(s)−exp(−s)}/{exp(s)+exp(−s)}, $a_{1i}∼N(90,2)$, $a_{2i}∼N(90,2)$, $b_{1i}∼N(30,90)$, $b_{2i}∼N(50,90)$, $c_{1}=0.2$ and $c_{2}=1$.
3. The power function
$$g_{k}(\tau_{ij}|T_{i},I_{i}=k;\phi_{i})=\frac{1}{20^{c_{ki}}}(a_{ki}-b_{ki}){\left(-\tau_{ij}\right)}^{c_{ki}},$$
  where $a_{1i}∼N(90,2)$, $a_{2i}∼N(90,2)$, $b_{1i}∼N(30,90)$, $b_{2i}∼N(50,90)$, $c_{1i}∼\text{Uniform}[\frac{1}{3}-0.5,\frac{1}{3}+0.05]$, $c_{2i}∼\text{Uniform}[0.05,0.15]$.

The same group membership prediction steps as above are adopted. For a Weibull shape parameter of 10 and N=100 subjects per group for training, the AUC values for the longitudinal error variances $\sigma_{\epsilon }^{2}=1,5,10,25,50$ are 0.9805, 0.9707, 0.9671, 0.9562, and 0.9381 for the reverse Gompertz function, 0.9804, 0.9684, 0.9584,0.9421, and 0.9204 for the hyperbolic tangent function, and 0.9873, 0.9927, 0.9911, 0.9826, and 0.9681 for the power function, respectively. AUC values for other settings are similar.

### 3.4 Modifications of Indexing Event Time Predictions

The same setting as the previous section is adopted, except that the group memberships can be any classifications that are of scientific interest instead of being limited to dementia onset. Additionally, the group memberships are assumed to be known while the time to the indexing events, referred as the survival time, is to be predicted for the target subjects. Based on the estimated Weibull AFT model parameters, the mean survival time is calculate using left‐truncated Weibull distribution for each target subject with the corresponding censor time as the threshold [30]. The squared difference of this mean survival time from the true survival time is calculated as the prediction squared error (PSE). The median values of PSEs are summarized in Table 3 for each Weibull shape parameter and the number of training sample size per group. It shows that the PSEs are larger for larger Weibull variance corresponding to shape parameter 3 than smaller Weibull variance corresponding to shape parameter 10. The differences among different training sample sizes are relatively small, suggesting that N=100 per group is sufficient for the working AFT models with 11 parameters.

**TABLE 3:** Median prediction squared errors from the baseline Weibull AFT models for the target subjects.

	Weibull	N training per group		
Group	Shape	100	200	500
Group 1	10	0.4916	0.4616	0.4830
	3	4.4452	4.4907	4.1710
Group 2	10	0.4882	0.5189	0.5087
	3	3.6435	3.7404	3.7142

The mean survival time predictions are then modified by incorporating the longitudinal data information. We first draw 1000 random samples that are bigger than the corresponding censor time for each subject. The weights of the samples are formulated by the probability density functions of the longitudinal data for each draw. The modified mean survival time is calculated as weighted averages. Their squared differences from the true survival time are also summarized by their median PSEs. To compare the survival time predictions before and after modifications, we take the ratios as after over before, for example, a ratio of 0.60 indicating a 40% reduction in median PSE.

The ratios under correctly specified longitudinal models with respect to the covariates effects are displayed in Figure 5. The median PSE reductions are around 20% to 80%. Under otherwise the same settings, the reductions in median PSEs are smaller for larger longitudinal error variances because of less information. The reductions for Weibull shape 3 are more than for Weibull shape 10, suggesting larger Weibull variances have more room to be improved. The differences among different training sample are relatively small, suggesting N=100 per group is a good sample size for these settings. The reductions for Group 1 is more than for Group 2, especially for Weibull shape parameter 3. This is because that Group 1 longitudinal trajectories have more curvatures, which in turn provide more information about the indexing events. The results for incorrectly specified longitudinal models are similar with comparable PSE reductions hence not displayed, which suggests robustness of the method, except a much worse case for Group 1, Weibull shape 10, N=200, without covariate effects, and $\sigma_{\varepsilon }^{2}=1$. This suggests that when the longitudinal error variance is small, it is possible for the proposed method to be sensitive to model misspecifications.

**FIGURE 5:**
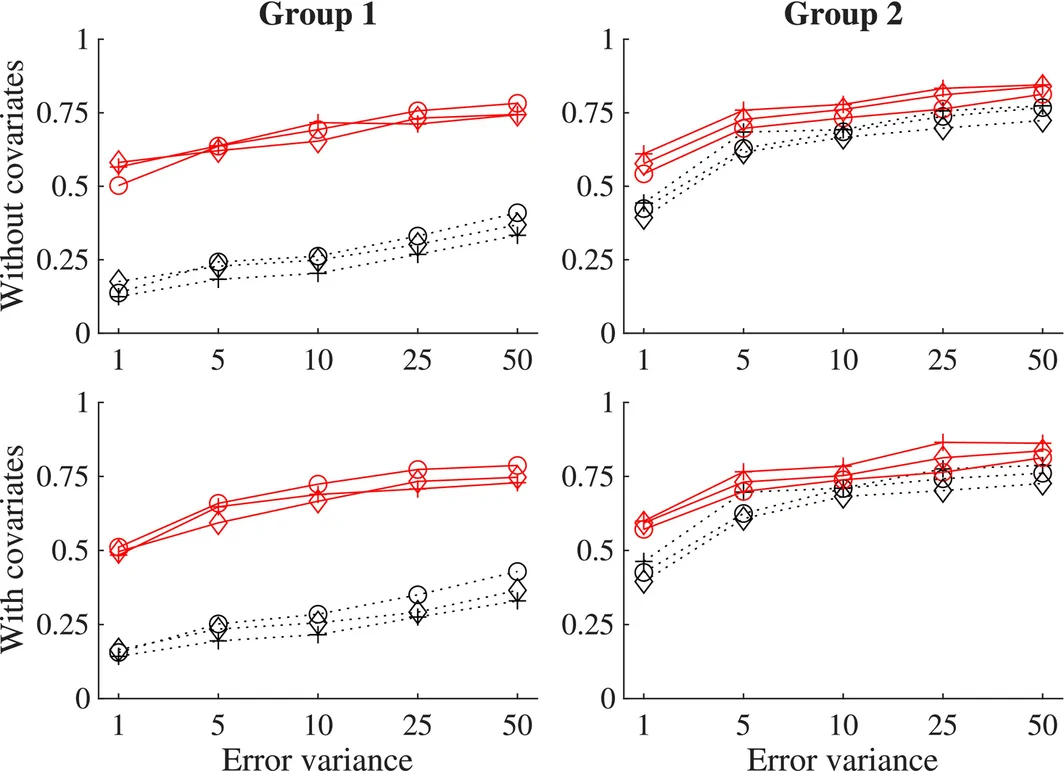
Simulation: Ratios of median PSEs as after over before incorporating the longitudinal data, whose models are correctly specified. The red solid lines are for Weibull shape parameter 10 while the black dotted lines are for shape parameter 3. Lines with plus signs are for N=100 training subjects per group, with circles for N=200, and with diamonds for N=500. The upper row is for the true models without and the lower row for the true models with the effects of years of education. The left column is for Group 1 while the right column for Group 2.

## 4 A Real Data Example

In this section, a real data example based on the Indianapolis‐Ibadan Dementia Project (IIDP) is used to illustrate how the proposed method can be used to predict group memberships in real world by right‐aligning partially observed longitudinal data to indexing events that have not happened. According to the study's website, IIDP “is a prospective population‐based comparative epidemiological study of the prevalence, incidence rates and risk factors for Alzheimer's disease and other age associated dementias.” Since its commencement in 1991, six waves of subjects, who are 65 years or older African Americans, have been enrolled from Indianpolis, Indiana, USA and Yoruba, Ibadan, Nigeria. Study subjects underwent regularly cognitive assessments and clinical evaluations for the diagnosis of dementia approximately every two or three years. Currently, IIDP has 7449 subjects with normal cognitive function, 302 subjects with cognitive impairments, and 778 subjects with dementia [4, 31].

We selected a subset of the IIDP subjects as our illustration data example from the Indianapolis cohort, enrolled in 1992 and 2001 with follow up data up to 17 years. The Community Screening Interview for Dementia (CSID) were obtained at baseline and year 3, 6, 9, 12, 15, and 17 after baseline. CSID has been widely used as a screening tool for dementia that evaluates multiple cognitive domains, including language, attention, memory, orientation, praxis, comprehension, and motor response. For this analysis, we used the CSID total score which incorporates all cognitive items and ranges from 0 to 80 [32]. We further required that the subjects died during the study follow‐up and had at least three longitudinal CSID total scores, which led to 433 subjects in total. Consequently, all the true indexing event time $T_{i}$'s and the group memberships were known, which were used to quantify the prediction performance of the proposed method and comparison methods. The baseline age range was 65 to 99 years with the median age as 75; 67% (291/433) were female; and 37% (161/433) were diagnosed with dementia before death. The numbers of CSID observations were 3 for N=188, 4 for N=135, 5 for N=60, 6 for N=34, and 7 for N=16 subjects.

To mimic real world situations, ten‐fold cross‐validation (CV) was used. For each fold, nine‐tenths of data were used as the training sets, while the other one‐tenth for prediction. For the target subjects of dementia prediction, their survival time and correspondingly their longitudinal data were uniformly censored over their longitudinal follow‐up durations. The group memberships and the time to the indexing events of these target subjects were thus assumed to be unknown. Their time to the indexing events was modeled and integrated out to predict whether they would develop dementia before death by the proposed method. The following methods were compared.

1. The proposed method.
  a. The group membership probabilities were both fixed as 0.50 and estimated from logistic regression models with covariates as baseline age, female, overweight, and years of education.
  b. Weibull regression models were adopted for the survival models using dementia status, baseline age, female, overweight, years of education, smoking, depression, hypertension, stroke, and diabetes.
  c. For the longitudinal data, the sigmoid mixed effects models in Equations ((6), (7), (8), (9), (10)), with and without the effects of years of education on the Hill slope parameters, were applied.
2. Baseline prediction models with predictors as baseline age, female, overweight, years of education, smoking, depression, hypertension, stroke, and diabetes.
  a. Random forest with 200 trees.
  b. Logistic models with two approaches.
    i. The same full model for all the 10‐fold CV.
    ii. For each CV fold, choose the best subset model.
  c. Fine and Gray [33]'s competing risk models [33].
    i. Dementia and death were separately modeled as the event of interest, which generated two sets of modeling fitting.
    ii. The cumulative incidence functions were calculated for the prediction sets for both dementia and death.
    iii. The median time‐to‐event was calculated and the differences of the median dementia time and the median death time were used to calculate AUC.
3. Lee et al. [5]'s “baseline and slope” model [5], which utilizes the longitudinal profiles after baseline.
  a. Same baseline predictors as the baseline prediction models.
  b. Fit linear mixed effects models with both fixed and random linear trends to the first three year CSID total score data, and obtain the best linear unbiased predictors (BLUPs) for the random slopes.
  c. The random slope BLUPs and baseline CSID total scores were added as additional predictors.

The logistic models and the random forest were adopted as baseline prediction models. Lee et al. [5]'s baseline and slop model was adopted to compare with longitudinal models. Fine and Gray [33]'s competing risk models were adopted to compare with predictions through modeling and predicting time‐to‐competing‐events.

Figure 6 shows the boxplots overlaid with swarm charts of the prediction probabilities with the prior group membership probabilities fixed at 0.50 and no years of education effects. It shows that although the results were not as good as those in the simulation, more than half of subjects from both groups had the correct prediction probabilities >0.55. Table 4 shows the AUC values from ROC analysis. For the proposed method, including effects of years of education lowered the AUC values, which suggests that years of education was not an important covariate and should not be included. Additionally, using the estimated prior group membership probabilities increased the AUC values by 0.0093 and 0.0112 for without and with the effects of years of education, respectively. The best AUC as 0.7215 was from the model without the effects of years of education but with estimated group membership probabilities, which was bigger than the AUCs from baseline prediction models by 0.0951–0.1522, and bigger than the AUC from Lee et al. [5]'s baseline and slope model by 0.0812. This approximately 10% increase in AUC values are in general considered as clinically meaningful.

**FIGURE 6:**
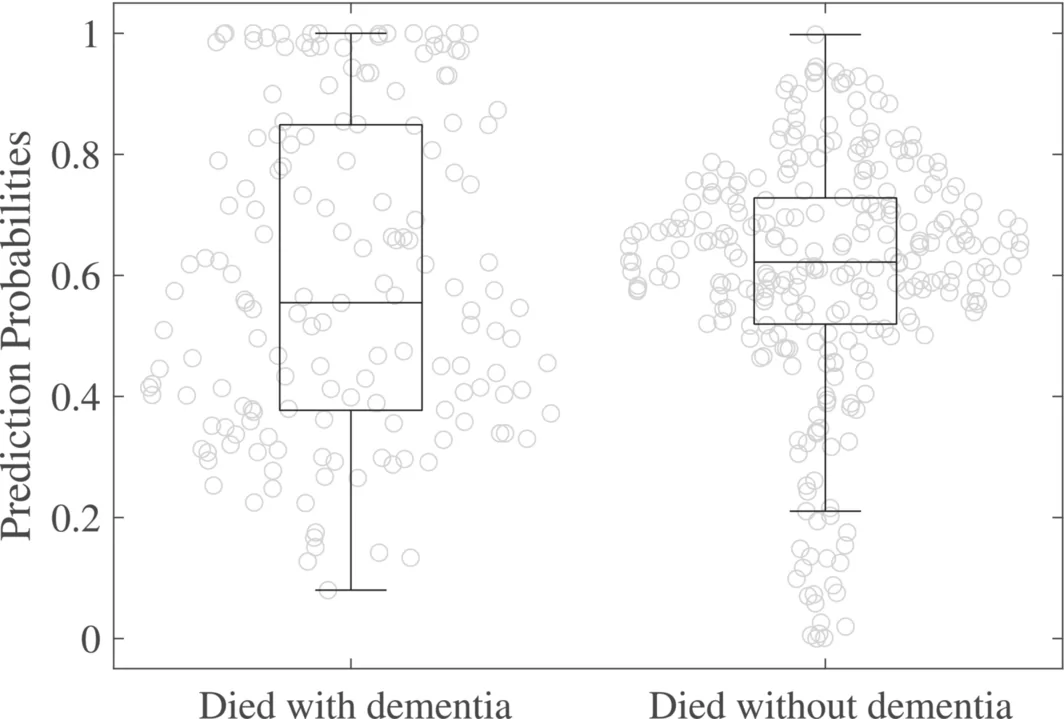
Data example: Prediction probabilities of belonging to the true groups by the proposed method with 10‐fold cross‐validation. Prior group membership probabilities were fixed at 0.50. Years of education were not included in the sigmoid mixed effects models.

**TABLE 4:** AUC values of the data example by 10‐fold cross validation.

Method	AUC
The proposed method	
Without effects of education Years	
Prior probabilities as 0.05	0.7122
Prior probabilities by logistic regression	0.7215
With effects of education Years	
Prior probabilities as 0.05	0.7095
Prior probabilities by logistic regression	0.7207
Baseline prediction models	
Logistic regressions	
Full model	0.6188
Best subset	0.6264
Random forest	0.5693
Fine and Gray's competing risk model	0.6213
Lee et al. [5]'s baseline and slope model	0.6403

SAS 9.4 was used in simulation for generating the baseline and survival data, and fitting Weibull regression models using PROC LIFEREG and logistic regression models using PROC LOGISTIC for both simulation and data analysis. Matlab R2024b function nlmefitsa, which implements the SAEM algorithms, was used for the longitudinal parameter estimation; and functions TreeBagger and predict were used for the random forest predictions.

Remark 10 The goodness‐of‐fit of the Weibull regression models to the survival data can be quantified by Harrell's concordance index (C‐index), which was 64.49% to 66.12% for the 10‐fold CV. These numbers were not optimal but close to the C‐indexes based on Cox proportional hazard models using the same set of predictors, with their differences between −0.09% and 0.09%. The goodness‐of‐fit of the longitudinal models can be quantified by marginal and conditional R‐squared, which were 0.25 to 0.27 for marginal and 0.64–0.67 for conditional R‐squared for the 10‐fold CV, respectively.

## 5 Discussion

In this article, we have developed a class of group‐specific nonlinear mixed effects models with right‐aligned indexing time to certain indexing events. These models are joint models of the group membership, the time to the indexing events, and the longitudinal data. Our simulation studies demonstrated that the method generated reliable group membership predictions and considerable error reductions in survival time predictions when the survival model part and the longitudinal model part were both correctly specified. When there were mild model misspecifications in the longitudinal models, the proposed method still generated reasonable predictions. In the real data example, the proposed method outperformed baseline prediction models with respect to AUCs by around 10%.

In the simulation, we have set the group membership probabilities to fixed values, and in the data example, we found the incorporation of the baseline dementia probabilities as optional. Baseline dementia prediction models have been widely studied for decades. For example, logistic regressions and survival models are widely used statistical models in dementia prediction [34, 35, 36, 37], and many machine learning methods are also used in dementia predictions [38, 39]. These baseline prediction models if correctly included may further improve the prediction performance.

The adoption of parametric but nonlinear mean functions for the longitudinal data was mainly based on their nonlinear curvatures, according to which an individual longitudinal profile will be aligned to group‐average profiles. In the literature of dementia modeling, polynomial change point mixed effect models [40], parametric nonlinear mixed effects models [3], and nonparametric curves [41, 42] had been adopted, which had been compared and concluded to have similar performance [19]. When the longitudinal data have nonlinear curvatures and the indexing time needs to be modeled, linear mixed effects models and change‐points mixed effects models are likely not flexible enough for the curvatures. Nonparametric models such as functional mixed effect models [43], on the other hand, do not have scientifically interpretable parameters. However, simulations studies may be needed to compare their finite sample performance, which is beyond the scope of this article.

The proposed method has several limitations. The use of SAEM entails greater computational demands, particularly for complex nonlinear mixed effects models and large datasets. Furthermore, when accurate finite‐sample inference is important, bootstrap‐based standard error estimation may be preferable to asymptotic standard errors. Bootstrap inference, however, requires repeated model fitting and therefore adds to the computational burden. This represents an important practical consideration rather than a limitation specific to the proposed modeling framework, as the additional computational cost can be weighed against the potential benefit of more reliable uncertainty quantification.

In addition, the proposed model used static baseline survival models. With the accumulating longitudinal data, the survival model could be modified to incorporate the longitudinal data. The group membership prediction concerns only whether a subject will develop a certain condition without considering the onset time, which may also be of critical clinical interest. When the time‐to‐grouping event, such as time to dementia diagnosis, is of interest and is also used to index time for the longitudinal data, a different model framework may need to be explored, as not all subjects are expected to develop the condition. The simulation and data example excluded random effects that are in the denominators in the mean functions, which is likely an oversimplification of the longitudinal processes. Including random effects in all the model parameters may be capable of characterizing the longitudinal functions more accurately, but their computational burdens will also increase significantly. We did not consider model selections for either the survival models or the longitudinal models. We did not compare with competing risk models with time‐varying covariates, because these modes are still active research topics with uncertain performance [44, 45]. In Section 2.2.1, we have required the availability of both indexing event time and group membership from a subset of subject. The availability of indexing event time of a subset of subjects is always necessary for the corresponding sub‐models. On the other hand, the availability of the group memberships of a subset of subjects could be relaxed, which will require clustering analysis of right‐aligned longitudinal profiles. In Section 2.3.3, we required the group membership to be known to modify the indexing event time predictions. This may be relaxed by first classifying the target subject to one of the groups and then modifying the index event time prediction while accounting for the classification uncertainties. We have relied on existing algorithms for parameter estimates by applying the SAEM algorithms in Kuhn and Lavielle 2005 [22]. Our simulation suggested that although the longitudinal parameters can be estimated with small biases from complete data, including missing group memberships and survival time may introduce significant biases. One potential reason is that Kuhn and Lavielle 2005's method was designed to handle random effects in longitudinal models without considering missing group memberships or survival time which serves as an indexing event for the longitudinal data. Extending the proposed models to address these limitations will be interesting future research topics.

## Funding

Dr. Gao's work was partially funded by NIA grants K07AG076659, P30AG072976, U24AG021886, and U01AG057195.

## Conflicts of Interest

The authors declare no conflicts of interest.

## Data Availability

The data that support the findings of this study are available from the corresponding author upon reasonable request.
